# Circadian rhythm disruption as a potential contributor to BPPV: Evidence from a young rat model vestibular effects of circadian disruption

**DOI:** 10.1371/journal.pone.0339869

**Published:** 2025-12-30

**Authors:** Hüsniye Gül Otlu, Hanifi Korkmaz, Nilüfer Diller Bulut, Nurcan Göktürk

**Affiliations:** 1 Vocational School of Health Services, Medical Laboratory Techniques Program, Malatya Turgut Ozal University, Malatya, Türkiye; 2 Vocational School of Health Services, Malatya Turgut Ozal University, Malatya, Türkiye; 3 Department of Nutrition and Dietetics, Malatya Turgut Ozal University, Malatya, Türkiye; 4 Department of Medical Biochemistry, Inonu University, Malatya, Türkiye; Belgrade University Faculty of Medicine, SERBIA

## Abstract

Circadian rhythm disturbances, increasingly common due to artificial lighting and modern lifestyle factors, may underlie vestibular dysfunction such as benign paroxysmal positional vertigo (BPPV), even in younger populations. This study aimed to investigate the effects of circadian rhythm disruption on balance performance and vestibular biomarkers in a young rat model. Young male Wistar rats were exposed to constant light (CL) for 4 weeks to induce circadian disruption, while control rats were maintained under a standard 12:12-hour light–dark cycle. Following the exposure, serum and cochlear tissues were analyzed for otolin-1, vitamin D3, melatonin, and electrolytes (Ca² ⁺ , Na ⁺ , K ⁺ , Cl⁻). Balance was evaluated using the rotarod performance test. Constant light exposed rats showed significantly elevated otolin-1 levels in both serum and cochlear tissues, along with reduced melatonin levels and impaired rotarod performance. Vitamin D3 levels were lower in the CL group, while serum electrolytes remained unchanged. Circadian rhythm disruption may impair vestibular function in young rats via melatonin related pathways or otolin-1 modulation, independent of serum electrolytes. Our results imply that circadian rhythm disruption may contribute to BPPV through pathways unrelated to aging or bone metabolism.

## Introduction

Benign paroxysmal positional vertigo (BPPV) is the most common peripheral vestibular disorder, characterized by brief episodes of vertigo triggered by changes in head position. The pathophysiology of BPPV is primarily associated with the displacement of otoconia from the utricle into one of the semicircular canals most commonly the posterior canal, but also the horizontal or anterior canals leading to inappropriate stimulation of the ampullary crista and the onset of vertiginous symptoms [1,2].

Recent research has identified Otolin-1 a glycoprotein component of the otoconial matrix as a promising circulating biomarker for inner ear disorders, particularly those involving otoconia degeneration such as BPPV. Otolin-1 is a collagen-like glycoprotein predominantly localized in the vestibular organs, including the utricle and saccule, where it contributes to the structural integrity of otoconia [3]. Under physiological conditions, the inner ear is protected by the blood-labyrinth barrier, a selective interface similar to the blood-brain barrier, which prevents the free passage of inner ear proteins into the systemic circulation. However, in certain pathological conditions such as aging, inflammation, or trauma this barrier may become compromised, facilitating the leakage of inner ear-specific proteins like Otolin-1 into the bloodstream [4]. Clinical studies have demonstrated significantly elevated serum Otolin-1 levels in patients with BPPV, supporting the hypothesis that otoconial degeneration or displacement may increase inner ear permeability [5]. Thus, the passage of Otolin-1 from the inner ear to the serum represents not only a marker of vestibular dysfunction but also a potential diagnostic tool for BPPV and related balance disorders.

Circadian rhythms are endogenous, approximately 24-hour biological cycles that synchronize with the external light-dark environment and regulate numerous physiological functions, including hormone secretion and sleep-wake cycles [6]. Disruption of circadian rhythms particularly through continuous light exposure can impair the secretion of neuroendocrine factors such as melatonin and cortisol, both of which are critical regulators of vestibular and autonomic function [7]. Recent evidence suggests that the vestibular system itself may be under circadian control, and that circadian dysregulation may negatively impact vestibular performance and balance [8].

In addition, vitamin D3 deficiency has been widely associated with both the onset and recurrence of BPPV. Clinical data support that individuals with low serum vitamin D3 concentrations are more susceptible to BPPV and experience higher recurrence rates, which can be mitigated with vitamin D supplementation [9]. In addition to epidemiologic links between low vitamin D and BPPV, mounting evidence suggests bidirectional crosstalk between circadian clocks and vitamin D signaling. In human adipose-derived stem cells, calcitriol (vitamin D3) can synchronize core clock gene expression (BMAL1, PER2), implying that vitamin D status may shape circadian outputs at the transcriptional level [10]. More recently, an in vivo intervention identified dozens of vitamin D target genes in immune cells that display circadian behavior and exhibit coordinated downregulation after vitamin D3 supplementation, highlighting a clock-sensitive vitamin D gene network [11]. Beyond vitamin D, electrolyte balance particularly sodium regulation plays a critical role in maintaining inner ear fluid homeostasis. High sodium intake has been linked to alterations in endolymphatic volume and pressure, which may exacerbate vestibular symptoms in susceptible individuals [12].

In recent years, a notable increase in the diagnosis BPPV among younger individuals has been reported [13]. While BPPV is classically associated with aging-related changes such as osteoporosis-induced alterations in mineral homeostasis and hormonal imbalances, these mechanisms may not fully explain its pathophysiology in the younger population. Instead, emerging evidence suggests that lifestyle-related circadian rhythm disruptions driven by factors such as night shift work, prolonged exposure to artificial lighting, and irregular sleep–wake cycles may play a critical role in the onset of vestibular dysfunction in this demographic. We hypothesize that circadian rhythm disruption may contribute to BPPV through mechanisms distinct from those in older adults. To test this hypothesis, the present study investigates the effects of continuous light exposure a well-established model of circadian rhythm disruption on serum and cochlear otolin-1 levels, serum vitamin D3 concentrations, and motor coordination performance in young rats. These findings may offer new insights into the relationship between circadian biology and vestibular pathophysiology and contribute to a deeper understanding of BPPV etiology beyond age-related mechanisms.

## Materials and methods

### Experimental design

Thirty male Wistar Albino rats (12 weeks old; 300–350 g) were obtained from the Inonu University Experimental Animal Production and Research Center. The study was approved by the Inonu University Animal Ethics Committee (Approval No: 2022/7–6). All animal experiments were conducted by the ARRIVE guidelines (Animal Research: Reporting of In Vivo Experiments) to ensure transparent and comprehensive reporting of in vivo studies [14].

**Fig 1:**
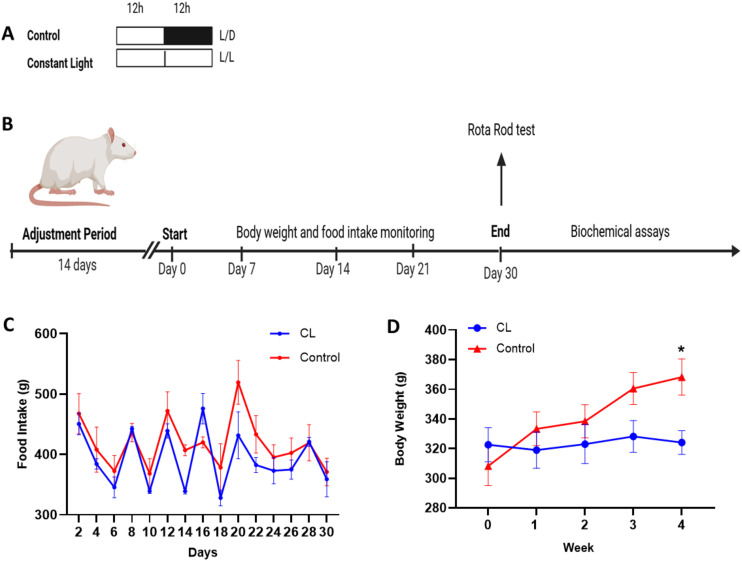
Effects of constant light exposure on body weight and food intake in rats. **(A)** Experimental timeline. Rats were randomly assigned to either the control group (12:12 h light/dark cycle, L/D) or the constant light group (24-hour light exposure, L/L) for 30 days. **(B)** Body weight and food intake were monitored regularly throughout the experiment. On Day 30, motor coordination was evaluated using the Rota Rod test, followed by euthanasia and sample collection for biochemical analyses. **(C)** Food intake was measured every two days per cage (n:5). Total food consumption was significantly lower in the CL group compared to the control group (**p:0.0102; n:15). **(D)** Initial body weights were similar between groups. By the end of the study, control animals exhibited a significant increase in body weight, whereas the CL group showed no significant change. At week 4, body weight in the control group was significantly higher compared to the CL group (*p = 0.0302; n:15). All data are presented as mean ± SEM.

All animals underwent a two-week acclimatization period prior to the experiment. During the 30-day study, the control group was maintained under standard 12-hour light/12-hour dark conditions at a room temperature of 25 ± 2 °C. In contrast, the constant light (CL) group was continuously exposed to light to induce a circadian rhythm disruption model [15] (Fig 1A). Animals were fed a standard pellet diet and had ad libitum access to water. Food intake and body weight were monitored periodically throughout the study (Fig 1B).

### Termination of the experiment and sample collection

At the end of the fourth week, rats were deeply anesthetized with ketamine (80 mg/kg, i.p.) and xylazine (10 mg/kg, i.p.). Adequate anesthetic depth was confirmed by loss of pedal-withdrawal and corneal reflexes and lack of response to a tail pinch. Animals were kept on a warming pad and ophthalmic lubricant was applied as needed. All animals were euthanized under deep anesthesia by exsanguination via cardiac puncture at 14:00; a secondary physical method (bilateral thoracotomy) was performed to ensure death. Blood samples were collected and centrifuged at 3000 rpm for 15 minutes at 4 °C to obtain serum. Subsequently, skulls were stabilized using a fixation apparatus, and the auricle and posterior auditory canal wall were surgically removed to expose the basal turn of the cochlea. Dissected cochlear tissues and serum samples were stored at –80 °C until biochemical analyses.

Cochlear tissues were homogenized in phosphate-buffered solution using a mechanical homogenizer. The homogenates were centrifuged at 12,000 × g for 10 minutes at 4 °C to remove cellular debris. Supernatants were collected and stored for Otolin-1 quantification using enzyme-linked immunosorbent assay (ELISA).

### Serum Vitamin D3 analysis

Serum 25-hydroxyvitamin D [25(OH)D] levels were determined using liquid chromatography–tandem mass spectrometry (LC-MS/MS) (Thermo Scientific Ultimate-3000). Sample separation was achieved on a C18 reversed-phase column (100 mm × 2.1 mm, 1.8 µm) using a gradient elution protocol with a mobile phase of methanol and water (0.1% formic acid). Detection was performed using a tandem mass spectrometer (API 4000, AB Sciex) with an electrospray ionization source in multiple reaction monitoring mode.

### Melatonin and Otolin-1 ELISA assays

Serum melatonin and both serum and cochlear Otolin-1 levels were measured using commercially available ELISA kits (Bioassay Technology Laboratory, China), according to the manufacturers’ protocols.

### Serum electrolyte analysis

Serum concentrations of sodium (Na⁺), potassium (K⁺), chloride (Cl⁻), and calcium (Ca²⁺) were measured using an automated biochemical analyzer (Beckman Coulter AU5800, USA) with an ion-selective electrode module. Spectrophotometric methods were used for quantification.

### Rota-Rod performance test

Motor coordination and balance were assessed using a Rota-Rod apparatus (Ugo Basile, Italy). Following a two-day training period (maximum 180 seconds on the rod), each rat underwent three consecutive test trials with increasing rotation speeds (4–40 rpm). The latency to fall (seconds) was recorded, with 10-minute rest intervals between trials to prevent fatigue.

### Statistical analyses

Power analysis was conducted to determine the required sample size. Based on an expected effect size of 0.8, a statistical power of 0.80, and an alpha level of 0.05, the required sample size was calculated.

Statistical analyses were performed using GraphPad Prism version 10.2 (GraphPad Software, Boston, MA, USA). The normality of data distribution was assessed using the Kolmogorov–Smirnov test. For normally distributed variables, including melatonin, serum electrolyte levels, otolin-1, vitamin D3, and behavioral test results, comparisons between two independent groups were conducted using the unpaired Student’s *t*-test.

## Results

### Body weight and food intake

Food intake was measured every two days per cage (n = 5). The total food consumption was significantly lower in the CL group (392.5 ± 12.2 g) than in the control group (418.1 ± 11.0 g) (p = 0.0102) (Fig 1C).

While initial body weights were comparable (CL: 322.7 ± 11.1 g; Control: 308.3 ± 13.15 g), by the end of the study, control animals exhibited a significant weight gain (368.3 ± 9.9 g), whereas the CL group showed no significant change (324.2 ± 8.0 g) (p = 0.0302) (Fig 1D).

### Otolin-1 levels in serum and cochlea

**Fig 2:**
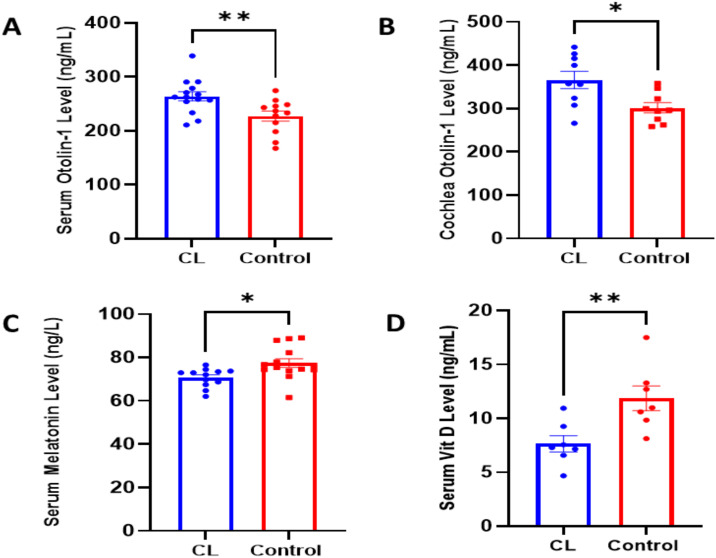
Biochemical effects of constant light exposure on Otolin-1, melatonin, and 25(OH)D levels in rats. **(A)** Serum Otolin-1 levels were significantly higher in the constant light (CL) group compared to controls, indicating a potential biomarker of otoconial disturbance (**p:0.0075; n:12-14). **(B)** Similarly, cochlear Otolin-1 concentrations were elevated in the CL group, supporting the possibility of inner ear structural changes under circadian disruption (*p:0.0129; n:9). **(C)** Melatonin levels, measured at the onset of the passive period, were significantly reduced in the CL group, confirming suppression of pineal activity and disruption of the circadian rhythm (*p:0.0113; n:12-14). **(D)** Serum vitamin D concentrations were markedly lower in CL-exposed rats, suggesting impaired synthesis or altered metabolism due to lack of dark-phase signaling (**p:0.0094; n:7). All data are expressed as mean ± SEM.

ELISA results showed significantly elevated serum Otolin-1 concentrations in the CL group (272.1 ± 7.6 ng/mL) compared to controls (222.8 ± 8.6 ng/mL) (p:0.0075) (Fig 2A). Similarly, cochlear Otolin-1 levels were higher in the CL group (366 ± 19.8 ng/mL) than in the control group (301.6 ± 11.7 ng/mL) (p:0.0129) (Fig 2B), suggesting possible otoconial degradation or displacement under constant light exposure.

### Altered melatonin secretion under constant light exposure

Melatonin concentrations were significantly reduced in the constant light (CL) group (70.7 ± 1.2 ng/mL) compared to the control group (77.5 ± 2.0 ng/mL) (p:0.0113) (Fig 2C). Melatonin analysis was performed on the blood samples collected at the beginning of the passive period. The observed suppression of melatonin in the CL group indicates a disruption of the normal circadian rhythm, likely reflecting a phase shift or dampening of rhythmic secretion. These findings confirm the effectiveness of the constant light protocol in inducing circadian rhythm disruption in this model.

### Constant light dampened Vitamin D3 in young rats

Serum vitamin D3 concentrations were significantly reduced in the CL group (7.63 ± 0.75 ng/mL) compared to the control group (11.87 ± 1.14 ng/mL), (p:0.0094), suggesting impaired vitamin D synthesis or metabolism under constant light exposure (Fig 2D).

### Serum electrolyte levels

**Fig 3:**
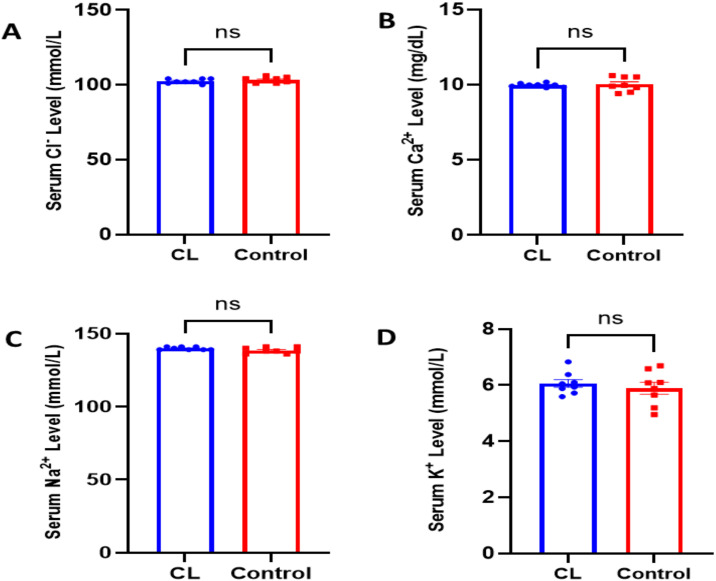
Assessment of serum electrolyte levels following constant light exposure. Serum levels of chloride **(A)**, calcium **(B)**, sodium **(C)**, and potassium (D) were measured to evaluate the impact of circadian rhythm disruption on electrolyte homeostasis. No statistically significant differences were observed between the CL and control groups (p > 0.05; n:8), and all values remained within normal physiological limits. These findings suggest that constant light does not markedly affect systemic electrolyte balance in young rats. Data are expressed as mean ± SEM.

In the circadian rhythm disruption model, electrolyte homeostasis was preserved in the young animals. No statistically significant differences were observed in serum electrolyte levels between the experimental and control groups. The levels of chloride (CL: 102.4 ± 0.53 mEq/L vs. Control: 103.3 ± 0.64 mEq/L; p = 0.310), calcium (CL: 9.96 ± 0.04 mg/dL vs. Control: 10.03 ± 0.16 mg/dL; p = 0.723), sodium (CL: 139.9 ± 0.35 mEq/L vs. Control: 138.5 ± 0.7 mEq/L; p = 0.1034), and potassium (CL: 6.06 ± 0.13 mEq/L vs. Control: 5.89 ± 0.21 mEq/L; p = 0.52) all remained within normal reference ranges. These findings suggest that electrolyte balance was maintained despite the circadian rhythm disruption (Fig 3A-D).

### Motor coordination – Rota Rod performance

**Fig 4:**
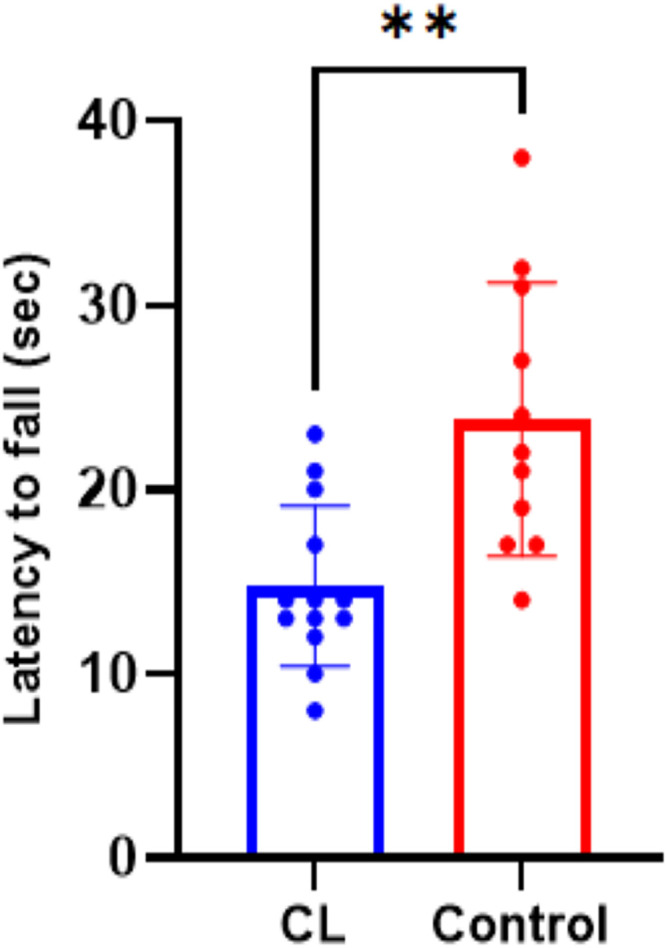
Rota Rod performance indicating impaired motor coordination in the CL group. The CL group showed a significantly reduced latency to fall compared to controls (*p* = 0.0014), suggesting vestibular dysfunction associated with circadian rhythm disruption. Data are expressed as mean ± SEM.

The CL group exhibited significantly poorer motor performance, as demonstrated by a shorter latency to fall (14.77 ± 1.2 s) compared to the control group (23.85 ± 2.2 s) (p = 0.0012) (Fig 4). This impaired motor coordination may indicate vestibular dysfunction linked to circadian rhythm disruption.

## Discussion

Modern lifestyles, particularly those involving night shift work and prolonged exposure to artificial light, have led to a growing prevalence of circadian rhythm disturbances. In this study, a rat model of circadian rhythm disruption induced by continuous light exposure was utilized to investigate the potential association with Benign Paroxysmal Positional Vertigo (BPPV), a vestibular disorder. Our findings demonstrate that this model significantly increases otolin-1 levels and impairs balance performance [16–18].

BPPV is more commonly observed in the elderly and is reported to be more prevalent among women. Several studies have linked BPPV with low serum vitamin D levels and calcium imbalances [19,20]. The biologically active form of vitamin D, calcitriol, plays a crucial role in maintaining calcium homeostasis and bone health by enhancing the absorption of dietary calcium and phosphorus [9]. Consistent with this biology, osteoporosis and vitamin D deficiency may compromise the otoconial matrix and increase the likelihood of crystal dislodgement from the utricle into the semicircular canals [21]. In addition, vitamin D signaling interfaces with circadian control: calcitriol can synchronize core clock genes, and many vitamin D target genes exhibit circadian behavior in vivo [10,11], pointing to bidirectional coupling between vitamin D status and clock function. Moreover, the use of young animals likely prevented osteoporotic changes, supporting the absence of alterations in calcium metabolism. In our experimental model, serum vitamin D3 was reduced in CL-exposed rats, whereas serum electrolytes (Ca² ⁺ , Na ⁺ , K ⁺ , Cl⁻) did not differ from controls. Because these animals lack cutaneous vitamin D synthesis and obtain vitamin D only from chow, the CL paradigm provides a sunlight-independent context in which circadian disruption itself may secondarily perturb vitamin D pathways. Although we did not quantify active vitamin D (calcitriol) or vitamin D metabolizing enzymes (e.g., CYP27B1, CYP24A1), a tissue-level functional vitamin D deficiency could still propagate circadian misalignment and destabilize the otoconial matrix despite normal systemic electrolytes [10]. By analogy, circadian misalignment in the CL setting could blunt or phase-shift cochlear/vestibular vitamin D signaling (e.g., VDR-regulated transcription), contributing to the observed rise in otolin-1 without detectable changes in serum calcium. Future work should profile active vitamin D, VDR-axis transcripts, and clock-gene phase in cochlear tissues, and test time-of-day–matched vitamin D3 rescue [11].

Circadian rhythm is an endogenous system that maintains a near-24-hour cycle, capable of persisting under constant light or darkness, while being synchronized to external light–dark cycles. Its period can vary between 20–26 hours [22]. Artificial lighting at night has been shown to disrupt this rhythm, causing oscillations in the secretion of several hormones [23]. Melatonin, a key regulator of circadian rhythm, exhibited significant changes in concentration under the disruption model, indicating successful model induction [24].

The vestibular system is known to be hormonally sensitive and expresses receptors for melatonin, cortisol, vasopressin, and sex hormones. Therefore, melatonin fluctuations may directly impact vestibular function [25]. One intriguing possibility raised by our findings is that otolin-1 expression may be regulated in a circadian-dependent manner. Otolin-1 mRNA is exclusively expressed in the inner ear, and its protein form is detectable in serum [3,26]. Rather than proposing direct clock-gene control of otolin-1, our findings are also compatible with an indirect pathway whereby circadian rhythm perturbations alter hormone and metabolic axes such as vitamin D status which in turn may promote otoconial/otolith damage and secondarily modify circulating otolin-1 levels. In this context, our data show that circadian disruption is associated with elevated serum otolin-1, motivating future studies to test whether clock-dependent changes in vitamin D and related pathways mediate these effects.

While the precise relationship between melatonin and otolin-1 remains to be elucidated, it is plausible that melatonin deficiency may upregulate otolin-1 expression through mechanisms independent of calcium homeostasis, such as increased oxidative stress, matrix remodeling, or epigenetic regulation. However, this possible interaction has not been directly studied, and more research is needed to understand the underlying mechanisms.

Due to the limited volume of endolymph, tissue analyses were performed on the entire cochlear tissue. The use of young animals may also explain the absence of detectable alterations in bone metabolism.

In conclusion, our findings raise the possibility that circadian rhythm disruption may play a role in the pathophysiology of BPPV. Future molecular studies are warranted to determine whether otolin-1 expression is regulated by circadian clock genes. Comparative studies involving different age groups, varying durations and intensities of rhythm disturbance, and detailed analyses of hormone receptor distribution in the vestibular system will further expand our understanding of this relationship. In our model, continuous light exposure was associated with significantly elevated otolin-1 levels in both serum and cochlear tissue, along with impaired balance performance. Notably, serum electrolytes (Ca² ⁺ , Na ⁺ , K ⁺ , Cl⁻) were unchanged, whereas vitamin D was reduced in CL-exposed animals; thus, circadian disruption may affect vestibular biology through vitamin-D linked and other non-electrolyte pathways. These findings suggest that BPPV risk may be influenced not only by aging but also by modern environmental factors such as aberrant light exposure, while underscoring the need for targeted mechanistic studies to establish causality.
